# *Canada Communicable Disease Report*—50 years later

**DOI:** 10.14745/ccdr.v50i05a01

**Published:** 2024-05-24

**Authors:** Michel Deilgat

**Affiliations:** 1 Office of the Chief Science Officer, Public Health Agency of Canada, Ottawa, ON

**Keywords:** *Canada Communicable Disease Report*, CCDR, Editor-in-Chief, infectious diseases

The first issue of *Canada Diseases Weekly Report* was published on May 10, 1975. The first article was entitled, “Outbreak report of staphylococcal, food poisoning in Northern Alberta.” I was about 14 years old when I filled out a subscription to the weekly report. Every week, I received in the mailbox an envelope with three folded, printed pages. I kept those pages in a binder which I carried with me over many, many decades. I could have never imagined then that 50 years later, I would be in the seat of the Editor-in-Chief of the *Canada Communicable Disease Report* (CCDR).

At its inception, the *Canada Diseases Weekly Report* was published by the Laboratory Centre for Disease Control, at Health and Welfare Canada. The report represented a major attempt to rapidly disseminate disease control information to Canadians across the country. It was rooted in the Epidemiological Bulletin, a monthly report introduced and published in the mid-fifties by the *Canadian Medical Association Journal*. The *Canada Diseases Weekly Report* specialized in disease surveillance, epidemiological investigations, case histories, international health, immunization information, and other activities in the realm of disease control.

Dr. Franklin M. M. White was appointed its first Editor and Eleanor Paulson Assistant Editor. In 1979, Ms. Paulson became the Managing Editor and finally, almost 10 years later, she became the Editor. In January 1992, the first issue of CCDR, formerly entitled *Canada Diseases Weekly Report* was published, always specializing in “disease surveillance, outbreak investigation, tropical health, and quarantine information, childhood immunization, infection control, sexually transmitted disease, and other disease control activities.” By 2001, having spent close to 30 years working for the journal, Eleanor Paulson became the Editor-in-Chief. Over the following years, several people took on the roles or duties of Editor-in-Chief or Managing Editor. Between 2009 and 2012, however, the journal became moribund, publishing supplements and a few Advisory Committee Statements (ACS) sporadically. Following the SARS outbreak, Dr. Ken Scott, senior medical advisor at the Infectious Disease Prevention and Control Branch, was appointed by Dr. Rainer Engelhardt to revive CCDR after several healthcare professionals and even a journalist inquired why the Public Health Agency of Canada was not publishing CCDR anymore. Dr. Scott took on the assignment and since he knew that Dr. Patricia Huston had previous experience in publishing, they both tackled this special project aiming to revive CCDR. During that time, all centres provided funding to support the journal. Dr. Engelhardt contributed with the working hours of one of his executive assistants for CCDR and that’s how it began!

In November 2013, Dr. Huston was appointed Scientific Editor with a short article entitled, “CCDR is changing”, announcing that the Public Health Agency of Canada’s flagship publication on infectious diseases was being revitalized as a biweekly issue with “Briefs” and useful links. In 2015, CCDR became a monthly issue interspersed with six supplements. The journal had also introduced a masthead and a 12-member Editorial Board by June 2015. A year later, the journal was completely redesigned, going from a Word PDF to a formal desktop-published version with a journal cover. In the following years, the quality of the journal kept improving both in design and content. In September 2018, the journal was accepted into PubMed, and in 2020, into the Directory of Open Access Journals, certified with a “DOAJ Seal.” In October 2019, I became the Editor-in-Chief and Dr. Huston became the Editor Emeritus, in recognition of all her exceptional contributions after almost six years at the helm of CCDR.

Today, the journal provides a platform to showcase and publish the work of the very diverse and specialized programs of the Public Health Agency of Canada, from the various branches at the Agency involved in infection prevention and control, including ACS from the National Advisory Committee on Immunization and reports from the Canadian Public Health Laboratory Network. CCDR publishes epidemiologic studies, eyewitness reports, implementation science research, outbreak reports, overviews, qualitative studies, rapid communication, surveillance reports, commentaries and several other types of articles. CCDR also publishes selected articles from the provincial, regional and local public health units, Canadian universities, and infectious disease departments from various hospitals across the country.

Today, CCDR and the *Health Promotion and Chronic Disease Prevention in Canada: Research, Policy and Practice* (HPCDP) journal are the two main, bilingual, peer-reviewed and open access scientific journals of the Public Health Agency of Canada. Together, we are proud to disseminate top quality Canadian data and findings to support evidence-informed discussions. We sincerely hope that our daily efforts contribute to informing, guiding and shaping public health actions, for the benefit of Canada and beyond.

